# Editorial: Consumer behavior around food safety and quality in the context of technological innovation

**DOI:** 10.3389/fnut.2024.1440242

**Published:** 2024-08-14

**Authors:** Shah Fahad, Shiyong Zheng, Fang Su, Giuseppe Antonio Di Vita

**Affiliations:** ^1^Business School, University of Jinan, Jinan, China; ^2^College of Digital Economics, Nanning University, Nanning, Guangxi, China; ^3^School of Economics and Management, Northwest University, Xi'an, China; ^4^University of Catania, Catania, Italy

**Keywords:** consumer behavior, food safety, technological innovation, public health issue, safety and quality

## Introduction

Food safety is at the center of human wellbeing in modern societies. Nowadays, food safety has become an important public health issue and therefore has been widely discussed in the academic world. In academia, the complex issue of food safety has been studied by different researchers, each with their own terminology and categorization. For example, Tirado et al. examined how climate change and variability affect the occurrence of food safety hazards at various stages of the food chain from primary production to consumption (1); Nayak and Waterson examined the food safety culture of a number of industry stakeholders in the United Kingdom (2); Wongprawmas and Canavari provide categories of food safety labeling as an indicator of consumers' willingness to pay in Thailand, and so on (3). Despite the growing academic interest in food safety issues in recent years, food safety incidents remain problematic and have not decreased significantly over the past decade.

In today's rapidly evolving field of technological innovation, there is a paradigm shift in consumer behavior around food safety and quality. As the global food supply chain becomes more interconnected and complex, technological innovation plays an increasingly critical role in ensuring the safety and integrity of the food we consume.

Among the published Research Topics, a number of forward-looking studies have responded positively to the current call from academia to push the frontiers of research literature in the field with their unique insights and contributions. Here, we review these cutting-edge papers and suggest future possibilities for theoretical and practical developments.

The focus of this Research Topic is to explore consumer behavior toward food safety and quality in the context of technological innovation and also considers climate change perceptions and the impact of environmental factors on nutrition and food safety. We solicited creative and innovative submissions focusing on, but not limited to, the following Research Topics:

- The extent to which food safety influences theories of consumer behavior.
- Influence of social media marketing on the buying attitudes of businesses and consumers, including “Netflix” economy and food products.
- Evolution of buyer-seller network dynamics as a result of food safety.
- Repercussions faced by companies and consumers due to the emergence of new and innovative food products in the post-epidemic era.
- Meta-analyses of research into the impact of consumers' health and illness on their food-purchasing behaviors.
- The impact of food and agricultural policies and subsidies on food composition and quality.
- The impact of climate, agricultural, and energy policies on human nutrition and consumers' dietary shifts.
- Health inequalities, socio-economic status and diet quality in relation to nutrition.
- Impacts of environmental-oriented behaviors for food security in buyer-seller relationships.

## Overview of the Research Topic

The aim of this Research Topic is to explore *Consumer behavior around food safety and quality in the context of technological innovation*. Therefore, all the papers included in this Research Topic elaborate on the scope of research on food safety and quality, and consumer behavior in the context of technological innovation.

Ruan et al. examine the impact of agricultural insurance on consumer food safety based on empirical evidence from provincial data in China. The results show that agricultural insurance significantly reduces the incidence of foodborne diseases and improves food safety. The moderating effects test shows that agricultural insurance effectively promotes food safety through two key pathways: promoting agricultural technology innovation and reducing environmental pollution. In addition, moderating effects analyses indicate that increased consumer confidence is enhancing the impact of agricultural insurance (Ruan et al.).

Su and Wang explored packaging colors to seduce and influence consumers' perceptions and have a significant impact on product identification. Consumer expectations, experiences and behaviors are influenced through the manipulation of external packaging. The psychological literature on color and emotion is used as a basis to explore the effects of food packaging color and food type on consumer purchase intentions. This research will assist marketers in exploring the possibilities of packaging color to influence the physiological and cognitive dimensions of food-related consumer behavior (Su and Wang).

Maojie explored the ability of anchor characteristics (interactivity, professionalism, and popularity) to influence consumers' perceived value and increase their willingness to purchase at a premium. The findings reveal the key role of anchors in consumers' decision-making process for paying for Netflix food (in the realm of the food industry, Netflix Foods has relied on social media to explode on the internet and become a newcomer in the food industry; Maojie).

Bayır et al., who conducted experimental research based on rational behavior theory, explored the impact of understanding and attention on the safety of food additives on consumers' potential behavior intentions. Therefore, the results provided in this study have a multiplier effect on health agencies and organizations issuing digital health statements/reports on food supplements, developing sustainable consumption patterns, or guiding them in the use of organic food supplements (Bayır et al.).

As such, these articles fill a gap in the literature in the relevant field by providing an overview of research on the Research Topic of consumer behavior in relation to food safety in the context of technological innovations in relation to stakeholders, in addition to presenting a holistic perspective on agriculture, climate and other aspects related to it, highlighting the interconnectedness and dependence of these aspects.

## Author contributions

SF: Writing – review & editing, Conceptualization. SZ: Writing – original draft. FS: Writing – review & editing. GA: Writing – review & editing.
